# Management of everolimus-associated adverse events in patients with tuberous sclerosis complex: a practical guide

**DOI:** 10.1186/s13023-017-0581-9

**Published:** 2017-02-15

**Authors:** Mark Davies, Anurag Saxena, John C. Kingswood

**Affiliations:** 1 Department of Oncology, South West Wales Cancer Centre, Singleton Hospital, Swansea, SA2 8QA UK; 2 Division of Cancer and Genetics, Cardiff University School of Medicine, Institute of Medical Genetics, Cardiff, CF14 4XN UK; 3 Sussex Kidney Unit, Royal Sussex County Hospital, Eastern Road, Brighton, BN2 5BE UK

**Keywords:** Everolimus, Adverse events, Tuberous sclerosis complex, TSC, Subependymal giant astrocytoma, SEGA, Renal angiomyolipoma, AML

## Abstract

Tuberous sclerosis complex (TSC) is a genetic disorder characterised by highly variable comorbid dysfunction and subsequent morbidity. The mTOR inhibitor everolimus is indicated for the treatment of adult TSC patients with renal angiomyolipomas (AMLs) and for subependymal giant astrocytoma (SEGA) in both adults and children, based on data from the EXIST-1 and EXIST-2 trials. However, due to the historical predominance of everolimus in the oncology setting, some physicians who treat TSC patients may be unfamiliar with everolimus-associated adverse events (AEs) and appropriate management strategies. This article aims to serve as a resource for specialists including nephrologists, paediatricians, neurologists and geneticists who require practical guidance on the management of events such as non-infectious pneumonitis, rash, stomatitis, infections, and renal AEs. Additional consideration is given to drug interactions, hepatic impairment, fertility, and sexual maturation. Since patients with TSC receive clinical benefit from continued therapy, it is important that everolimus-related events are dealt with appropriately through strategies such as dose modification, interruption, the provision of supportive care, regular monitoring, and patient education.

## Background

### Tuberous sclerosis complex

Tuberous sclerosis complex (TSC) is an autosomal dominant genetic disorder [1]. Prevalence rates vary, but estimates typically fall in the range of 6.8 to 12.4 per 100,000 people [2]. TSC is caused by mutations in *TSC1* on chromosome 9 or *TSC2* on chromosome 16; however, two-thirds of cases result from *de novo* mutations [3]. Mutations in *TSC1* or *TSC2* result in inappropriate mTORC1 signalling within cells, and this is thought to be responsible for many of the features of TSC [4].

TSC is a highly variable condition in both the type and severity of its manifestations. A history of seizures has been reported in up to 85% of patients, often beginning in the first few years of life (>80% of patients) [5–7], with intellectual disability found in ~45% of cases [8]. The condition is also commonly associated with the development of benign tumours in organs such as the kidneys, brain, heart and skin [9, 10]. Renal angiomyolipomas (AMLs) are among the most common features of TSC [9, 10], affecting around ~70% of patients [11, 12]. They manifest as benign tumours composed of abnormal blood vessels and cells, with either adipocyte-like or smooth muscle-like phenotypes [9], and commonly occur in both kidneys [9]. Around 20% of patients with TSC develop subependymal giant astrocytoma (SEGA), a benign glioneuronal brain tumour [13], and ~10% of women with TSC develop symptomatic lymphangioleiomyomatosis (LAM), a condition characterised by cystic destruction of the lung, pneumothorax and chylous pleural effusion [1]. This comorbid organ dysfunction and associated disease burden [10] requires careful management. The care of people with TSC can therefore be complex and requires a multidisciplinary approach [14].

### Treating TSC

Normalisation of defective mTORC1 signalling may be an effective approach to managing patients with TSC. Several mTORC1 inhibitors are in clinical use for many conditions and have the potential as therapeutic agents in TSC. Clinical trials have demonstrated the efficacy of some of these agents in the treatment of TSC. The mTOR inhibitor sirolimus has shown efficacy in clinical trials investigating the treatment of patients with AML and LAM [15–18], while another mTOR inhibitor, everolimus, is indicated for the treatment of adult patients with renal AML associated with TSC who are at risk of complications (based on factors such as tumour size or presence of aneurysm, or presence of multiple or bilateral tumours), but who do not require immediate surgery; and of patients with SEGA who require therapeutic intervention, but who are not amenable to surgery [19]. Three randomised, placebo-controlled phase III trials have demonstrated the efficacy and safety of everolimus in these patients [20–22].

EXIST-1 assessed the efficacy and tolerability of everolimus 4.5 mg/m^2^/day compared with placebo in 117 patients with a SEGA of ≥1 cm in diameter, and showed that response rate (reduction in the total volume of all target SEGA of 50% or more relative to baseline, in the absence of worsening of non-target SEGA, new lesions of 1 cm or greater in diameter, and new or worsening hydrocephalus) was greater in everolimus- than placebo-treated patients (35% versus 0%; *p* < 0.0001) [21]. At a median follow-up of 9.7 months, 97% of patients in the everolimus group were undergoing treatment; furthermore, no everolimus-treated patients discontinued due to disease progression. In an open-label extension phase of EXIST-1, responses persisted in 94% of responders, and only 8% of all patients had SEGA progression at a median follow-up of 28.3 months [23]. EXIST-2 compared oral everolimus 10 mg/day with placebo in 118 adults with TSC and at least one AML of ≥3 cm in diameter [20]. In this study, 42% of everolimus- and 0% of placebo-treated patients (*p* < 0.0001) had a confirmed AML response (reduction in AML volume [sum of volumes of all target AMLs identified at baseline] of ≥50% or more relative to baseline and absence of AML progression), with a median time to response of 2.9 months in the everolimus group [20]. Long-term follow up (median 28.9 months) showed an increase in response rate to 54%, with 97% of patients experiencing a reduction in tumour volume [24]. The final four-year analysis of 112 patients, including those who had crossed over from placebo, showed an overall response rate of 58% after a median duration of everolimus exposure of 204.1 weeks [19]. Recent data from the phase III EXIST-3 showed that adjunctive everolimus in patients receiving one to three antiepileptic drugs significantly reduced the rate of seizures in TSC patients refractory to treatment (29.3% and 39.6% reduction in seizure rate for 3–7 ng/mL and 9–15 ng/mL trough concentrations vs a 14.9% reduction with placebo), and increased the response rate (defined as ≥50% reduction; 28.2% and 40%, vs 15.1% for placebo) [22].

#### Safety of everolimus

The tolerability of everolimus in the EXIST studies was similar to that for other indications such as renal cell carcinoma, breast cancer, and transplantation [25–28]. In EXIST-1, 96 and 90% of everolimus- and placebo-treated patients, respectively, experienced AEs [21]. Common adverse events (AEs) seen in EXIST-1 and -2 are listed in Table 1 [20, 21]. In both studies, the majority of AEs were grade 1–2 in severity [21, 24], although in EXIST-1 grade 3–4 AEs were reported in 33 and 23% of patients in the everolimus and placebo groups, respectively, with stomatitis, pyrexia and convulsion being the most common such events [21]. A total of 2 grade 4 events occurred in EXIST-1 (gastroenteritis and hyperuricaemia) and 4 occurred in EXIST-2 (raised blood uric acid, neutropenia, seizure and hypertension); no patients died in EXIST-1 while one patient in EXIST-2 died from status epilepticus not related to treatment [20, 21].

**Table 1:** The most common adverse events (>10%) in SEGA patients aged 0–65 years in EXIST-1 and adult AML patients in EXIST-2 [20, 21]

Adverse event (all grades)	EXIST-1		EXIST-2	
	Everolimus 4.5 mg/m^2^ per day	Placebo	Everolimus 10 mg/day	Placebo
Mouth ulceration	32	5	16	5
Stomatitis	31	21	48	8
Convulsion	23	26	-	-
Pyrexia	22	15	-	-
Nasopharyngitis	18	23	24	31
Vomiting	17	13	15	5
Upper respiratory tract infection	15	18	10	5
Fatigue	14	3	18	18
Cough	13	10	20	13
Diarrhoea	13	5	13	5
Rash	12	5	-	-
Bronchitis	10	10	-	-
Otitis media	10	5	-	-
Pharyngitis	10	3	-	-
Acne-like skin lesions	-	-	22	5
Headache	-	-	22	18
Hypercholesterolaemia	-	-	20	3
Aphthous stomatitis	-	-	19	10
Nausea	-	-	16	13
Urinary tract infection	-	-	15	15
Anaemia	-	-	13	3
Arthralgia	-	-	13	5
Abdominal pain	-	-	11	8
Blood lactate dehydrogenase increased	-	-	11	5
Hypophosphataemia	-	-	11	0
Eczema	-	-	10	8
Leucopenia	-	-	10	8
Oropharyngeal pain	-	-	10	10

Female fertility-related AEs were a common finding in the EXIST-1 and -2 trials. In a pooled analysis of the data from these two studies (as well as a single-arm phase II study), the most common of such events were amenorrhoea (24.1%) and irregular menstruation (17.0%); the majority of amenorrhoea events were grade 1 or 2 in severity and resolved without intervention [29]. 6.3% of patients experienced grade 3 amenorrhoea. Everolimus had no effect on the timing of menarche in patients attaining menarche during the treatment period [29].

During long-term follow-up of EXIST-1, there were no differences in standard deviation scores for growth variables (height, height velocity, weight and weight velocity) in patients aged <18 years before and during treatment [23]; in the long-term follow-up of EXIST-2, glomerular filtration rate (GFR) remained stable [24], renal AEs occurred less frequently with everolimus than placebo [24], and no patients experienced angiomyolipoma-related bleeding [30]. In EXIST-3, everolimus exhibited a similar AE profile, with the most frequently reported events being stomatitis, diarrhoea, mouth ulceration, nasopharyngitis, upper respiratory tract infection, aphthous ulcer and pyrexia. Discontinuations due to AEs were low [22].

#### Everolimus dosing

Everolimus is available as either tablets or dispersible tablets, the availability of which differs from country to country. For patients with TSC who are unable to swallow tablets, the tablets can be completely dissolved in a glass containing ~30 mL of water through gentle stirring for approximately 7 min, immediately prior to drinking. Any remaining residue after drinking must be re-dispersed in ~30 mL of water and swallowed. Dispersible tablets dissolve in water more quickly than standard tablets.

Doses that are effective and well-tolerated vary between patients. Therefore, in order to obtain the optimal therapeutic effect, careful dose titration may be required. Treatment should also continue for as long as patients receive a clinical benefit, or until the case of unacceptable toxicity.

The recommended starting dose of everolimus in adult TSC patients with renal AML is oral 10 mg/day, and was used in the EXIST-2 and oncology studies, selected in order to maximise the probability of observing a therapeutic effect. Dose reductions and/or temporary interruption may be required in some patients who experience treatment-related AEs [19]; however evidence suggests that this may not impact therapeutic effect. There was no correlation between absolute change from baseline in blood concentration and the therapeutic effect in EXIST-2, other than patients with the highest trough concentrations having faster initial AML shrinkage [31], and despite the fact that a third of patients received dose reductions to 5 mg or 2.5 mg daily almost all patients who remained on everolimus experienced sustained and progressive AML shrinkage [24]. Hence, in clinical practice, many clinicians may use a starting dose of 5 mg in renal AML and titrate up or down according to therapeutic response and AEs. The dose can be titrated to achieve a trough concentration of 5–15 ng/mL; however, responses have been seen at trough concentrations as low as 3 ng/mL [20], so dose increases may not be necessary once acceptable efficacy has been achieved. Response of renal AML should be assessed after approximately 12 weeks of therapy by imaging of the kidneys. How frequently subsequent scans should be carried out is unclear. For patients who have been established on everolimus for some time and who are clinically stable, performing a scan at least annually is reasonable [14].

For SEGA, the dosing of everolimus should be individualised according to body surface area (BSA) using the Dubois formula, based on weight (W; in kg) and height (H; in cm) [19]:

$$ \mathrm{B}\mathrm{S}\mathrm{A} = {\mathrm{W}}^{0.425} \times {\mathrm{H}}^{0.725} \times 0.007184 $$

The recommended starting dose in SEGA is 4.5 mg/m^2^/day. A higher starting dose of 7 mg/m^2^/day could be considered for patients aged 1 year to <3 years. SEGA volume should be evaluated approximately 3 months after commencing everolimus, with subsequent dose adjustments due to changes in SEGA volume, corresponding trough concentration, and tolerability taken into consideration.

Everolimus is not licensed for the treatment of children with TSC-associated AMLs, but there is evidence of its effectiveness [32] and it is funded by NHS England [33]. If everolimus is used for non-licensed indications in children then the recommended dose to treat these cases can be drawn from the treatment of SEGA, where the recommended starting dose for children aged ≥3 years is 4.5 mg/m^2^/day. Everolimus is licensed and used worldwide for SEGA not amenable to surgery in both children and adults.

### TSC treatment: current challenges

There are a number of important challenges relating to everolimus use for the treatment of TSC. In particular, although everolimus is indicated for TSC-related AML and SEGA, the majority of experience with the drug comes from oncological indications. Furthermore, there is no clear and defined management pathway for TSC and patients may be seen by any number of different specialists depending on their presentation. In addition, many healthcare professionals who treat TSC are unfamiliar with everolimus and are unsure about prescribing it to their patients. AEs may be of particular concern; however, their successful management will increase the number of patients who can tolerate everolimus long-term and, therefore, help to optimise treatment benefit, which will help ensure sustained efficacy and adherence.

The aim of this article is to provide practical guidance to nephrologists and other specialists such as paediatricians, neurologists and geneticists who treat patients with TSC, including providing advice on what to consider at a patient’s first consultation and the key investigations to perform. Guidance will also be provided on the management of AEs, including lessons from everolimus use in oncological indications, as well as recommendations for patient education.

### Initiating everolimus in patients with TSC

**Fig. 1:**
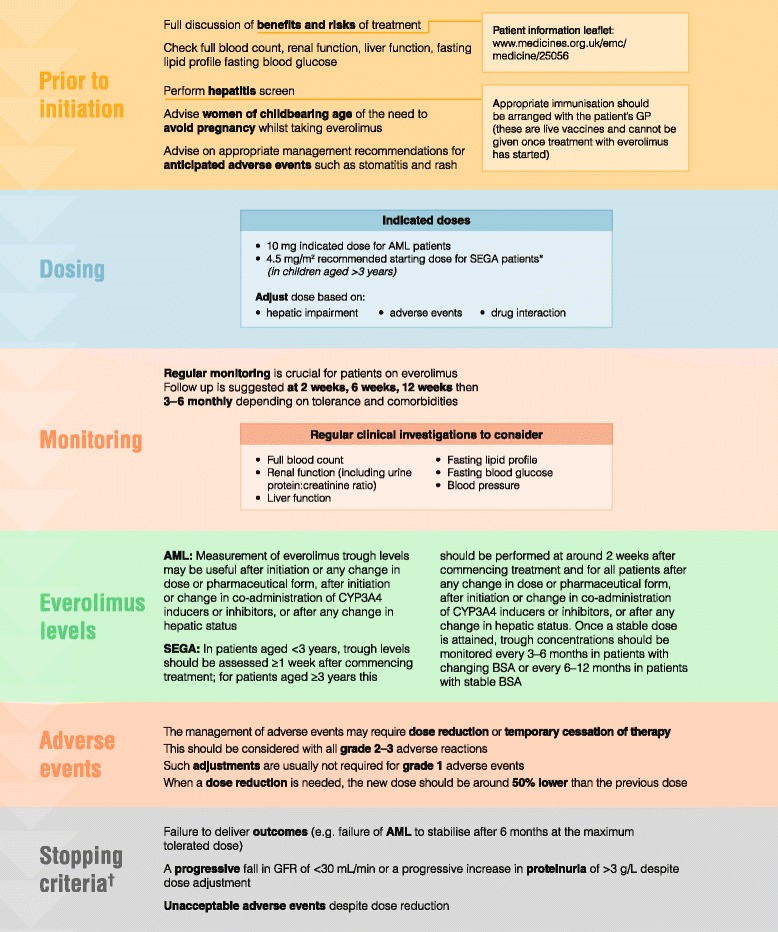
Protocol for initiation and monitoring of everolimus therapy. *≤1.2 m^2^ = 2.5 mg once daily, 1.3–2.1 m^2^ = 5 mg once daily, ≥2.2 m^2^ = 7.5 mg once daily; ^†^In the event of bleeding, temporary cessation of everolimus may be required for embolisation; everolimus may be restarted following healing if needed to control AML growth. *AML* angiomyolipoma, *BSA* body surface area, *GFR* glomerular filtration rate, *GP* general practitioner, *SEGA* subependymal giant astrocytoma

Patients with TSC require special considerations prior to the initiation of everolimus treatment (Fig. 1). Before treatment is given, a full blood count and liver and kidney function tests should be performed. Serum electrolytes and creatinine should be measured, and urine protein estimated by dipstick analysis and protein:creatinine ratio. Baseline lipids should be measured; if fasting lipids cannot be ascertained then random cholesterol is an acceptable substitute. If lipids are significantly elevated then improved lipid control should ideally be obtained before starting everolimus (e.g. through dietary modifications or statins).

Fasting blood glucose should also be measured, but in the absence of this a random glucose and glycated haemoglobin test can be performed. If these tests are abnormal, optimal glycaemic control should be achieved before starting everolimus whenever possible.

Because mTOR inhibitors have immunosuppressive properties, a thorough medical history relating to infection should also be obtained (e.g. history of pneumonia, recurrent otitis media, sinusitis, fungal infections, hepatitis, HIV, tuberculosis) [34]. A hepatitis screen may be warranted due to the potential risk of virus reactivation.

Patients with respiratory symptoms or radiological evidence suggestive of LAM should have a computed tomography scan, lung function tests, transfer factor of the lung for carbon monoxide and arterial oxygen saturation before everolimus administration.

Patients should be provided with information and advice on how their treatment may impact their daily life. It may be beneficial to pre-emptively prescribe or recommend items such as non-alcoholic mouthwashes and soft toothbrushes before the occurrence of AEs such as stomatitis. Another important consideration before prescribing treatment is to take an inventory of current medications patients are receiving; information on drug–drug interactions is presented later in this article.

Live vaccines should be avoided or completed before initiating everolimus owing to its immunosuppressive properties. Treatment with everolimus should be delayed or temporarily discontinued in patients undergoing surgery because the drug can delay wound healing [34]. Finally, as everolimus tablets contain lactose, patients with rare hereditary problems of galactose intolerance, Lapp lactase deficiency or glucose–galactose malabsorption should not take the drug [19].

### Paediatric considerations

Understandably, clinicians have concerns regarding the use of mTOR inhibitors in children, specifically regarding their effect on growth and gonadal function over the long-term. There are few studies that have evaluated the use of mTOR inhibitors in children with TSC [35, 36], however 5-year data from a phase II study show that growth parameters such as height, height velocity and weight in children were comparable before and after starting treatment [36]. Furthermore, paediatric kidney transplant studies with mTOR inhibitor-treated patients suggest no effect on longitudinal growth as well as comparable ages of sexual maturation and reproductive hormone levels when compared with kidney transplant patients who did not receive mTOR inhibitors [37–40]. Some studies of sirolimus have suggested the occurrence of testosterone suppression in adolescents [41] and adults [42–44], however most of these studies had small numbers of patients and used a higher dose regimen compared to recent studies.

Standard recommendation currently advises against concomitant use of everolimus on ketogenic diets in children with epilepsy, which is due to additive toxicity with hyperlipidaemia. Although data are limited regarding this side effect, the authors would recommend that clinicians use their discretion while prescribing mTOR inhibitors in such cases.

### Managing adverse events

Common AEs associated with the use of everolimus in TSC are stomatitis, nasopharyngitis, acne-like skin lesions, headache, cough and hypercholesterolaemia. Other AEs occurring in ≥10% of patients in the EXIST-1 and -2 trials are shown in Table 1 [20, 21]; the Common Terminology Criteria for Adverse Events (CTCAE) are shown in Table 2 [45].

**Table 2:** Grading of key everolimus-related adverse events based on National Cancer Institute Common Terminology Criteria for Adverse Events (CTCAE) [45]

Adverse event	Grade 1	Grade 2	Grade 3	Grade 4
Non-infectious pneumonitis	Asymptomatic	Symptomatic; not interfering with ADL	Severe symptoms; interfering with ADL, oxygen indicated	Life-threatening respiratory comprise; urgent intervention indicated
Infections	None	Localised; local intervention indicated	IV antibiotic, antifungal or antiviral intervention indicated; radiology/operative intervention indicated	Life-threatening consequences, e.g. septic shock, hypotension, acidosis, necrosis
Stomatitis	Minimal; normal diet	Symptomatic, but can eat and swallow; modified diet	Symptomatic; unable to adequately aliment or hydrate orally	Symptoms associated with life-threatening consequences
Rash	Macular or popular eruption or erythema without associated symptoms	Macular or papular eruption or erythema with pruritus or other associated symptoms; localised desquamation or other lesions covering	Severe, generalised erythroderma or macular, papular or vesicular eruption; desquamation covering ≥50% BSA	Generalised exfoliative, ulcerative, or bullous dermatitis
Metabolic events				
Hypercholesterolaemia,	>ULN–300	>300–400	>400–500	>500
mg/dL (mmol/L)	(>ULN–7.75)	(>7.75–10.34)	(>10.34–12.92)	(>12.92)
Hyperglycaemia,	>ULN–160	>160–250	>250–500	>500
mg/dL (mmol/L)	(>ULN–8.9)	(>8.9–13.9)	(>13.9–27.8)	(>27.8 or acidosis)
Hypophosphataemia,	<LLN–2.5	<2.5–2.0	<2.0–1.0	<1.0
mg/dL (mmol/L)	(<LLN–0.8)	(<0.8–0.6)	(<0.6–0.3)	(<0.3)
Hypertriglyceridaemia	>ULN–2.5 × ULN	>2.5–5.0 × ULN	>5.0–10.0 × ULN	>10.0 × ULN
	-	-	-	-
Hyperiuricaemia, mg/dL (mmol/L)	>ULN–10 (≤0.59 without physiologic consequences)	-	>ULN–10 (≤0.59 without physiologic consequences)	>10 (>0.59)
Myelosuppression, 10^9^/L				
Platelets	<LLN–75.0	<75.0–50.0	<50.0–25.0	<25.0
Neutrophils	<LLN–1.5	<1.5–1	<1.0–0.5	<0.5

*ADL* activities of daily life, *BSA* body surface area, *IV* intravenous, *LLN* lower limit of normal, *ULN* upper limit of normal

The management of AEs may require dose reduction or temporary cessation of therapy (Table 3) [19]. As a general rule, this should be considered with all grade 2–3 AEs thought to be everolimus treatment-related; adjustments are usually not required for grade 1 AEs. When a dose reduction is needed, it is recommended that the new dose should be around 50% lower than that previously administered. For dose reduction below the lowest available strength, alternate day dosing should be considered. If the AE is persistent or recurrent, the dose should be interrupted for 3–14 days (or until the AE is resolved to grade ≤1), with everolimus then restarted at a lower dose. Dose interruption/reduction can help to ameliorate AEs while allowing for continued therapeutic benefit, as evidenced in EXIST-2 in which 71% of patients had a dose interruption/reduction and around a third were maintained on doses less than the initial 10 mg/day [24].

**Table 3:** Dose modification recommendations for key everolimus-related adverse events

Adverse event	Grade 1	Grade 2	Grade 3	Grade 4
Non-infectious pneumonitis [19]	Consider a 50% decrease in everolimus dose	Consider interruption of therapy until symptoms improve to grade ≤1; re-initiate at 50% of previous dose. Discontinue if no recovery within 4 weeks	Interrupt everolimus until symptoms resolve to grade ≤1; consider re-initiating at 50% of previous dose. If toxicity recurs at grade 3, consider discontinuation	Discontinue everolimus
Infections^a^	No change in everolimus dose	Maintain dose if tolerated; interrupt if intolerable or grade 2 recurrence until recovery to grade ≤1 then restart at same dose.	Interrupt dose until recovery to grade ≤1, then restart at reduced dose. If dose interrupted >21 days, consider discontinuation	Discontinue everolimus
Stomatitis [19]	-	Temporary dose interruption until recovery to grade ≤1; re-initiate at same dose. If AE recurs at grade 2, interrupt dose until recovery to grade ≤1; re-initiate at 50% of previous dose	Temporary dose interruption until recovery to grade ≤1; re-initiate at 50% of previous dose	Discontinue everolimus
Rash^a^	-	If toxicity tolerable, no dose adjustment required. If intolerable, temporary dose interruption until recovery to grade ≤1; re-initiate at same dose. If AE recurs at grade 2, interrupt dose until recovery to grade ≤1; re-initiate at 50% of previous dose	Temporary dose interruption until recovery to grade ≤1. Consider re-initiating at 50% of previous dose. If toxicity recurs at grade 3, consider discontinuation	
Metabolic events [19] Hypercholesterolaemia Hyperglycaemia Hypophosphataemia Hypertriglyceridaemia Hyperiuricaemia	-	No dose adjustment required	Temporary dose interruption; reinitiate at 50% of previous dose	Discontinue everolimus
Myelosuppression [19] Platelets Neutrophils		Temporary dose interruption until recovery to grade ≤1; reinitiate at same dose No dose adjustment required	Temporary dose interruption until recovery to grade ≤1; reinitiate at 50% of previous dose Temporary dose interruption until recovery to grade ≤2; reinitiate at same dose	Temporary dose interruption until recovery to grade ≤1; reinitiate at 50% of previous dose Temporary dose interruption until recovery to grade ≤2; reinitiate at 50% of previous dose
Febrile neutropenia [19]	-	-	Temporary dose interruption until recovery to grade ≤2 (≥1.25 × 10^9^/L) and no fever; reinitiate at approximately 50% of previous dose	Discontinue everolimus

*AE* adverse event

^a^Author recommendation

Many patients with TSC have learning difficulties and may not be reliably able to report any toxicities, or the toxicities may manifest in atypical ways such as altered behaviour, e.g. pain can manifest as increased aggression, withdrawal or sleep disturbance. Family members and other carers are often attuned to such changes, and any concerns they raise must be taken seriously.

Based on lessons from clinical trials in TSC-related conditions and oncology indications, and from our own clinical experience, we have provided the following guide as a reference for nephrologists and other specialists who may encounter specific AEs in their patients. AEs of note have been listed, along with a summary of their presentation, practical advice for physicians and important information that should be communicated to patients to aid in their management.

### Non-infectious pneumonitis

Non-infectious pneumonitis is a class effect of rapamycin analogues, such as everolimus and temsirolimus. A diagnosis of non-infectious pneumonitis should be considered in patients with TSC presenting with new or worsening signs, or with non-specific respiratory symptoms such as cough or dyspnoea, the development of a pleural effusion, or radiological changes (ground-glass opacities and focal consolidation, predominantly in the lower lobes). Pneumonitis grading is shown in Table 2.

Recommendations for dose modification in everolimus-treated patients experiencing non-infectious pneumonitis are provided in Table 3. Differentiating non-infectious pneumonitis from infection is a key issue for investigations. *Pneumocystis jiroveci pneumonia* and *Legionella* should be ruled out.

Treatment interruption, dose reductions and treatment with corticosteroids (e.g. in adults, 3–7 days of 30–60 mg prednisolone followed by steroid tapering) and antibiotics are the common management strategies for non-infectious pneumonitis. It is important to try and exclude an infectious aetiology before the use of corticosteroids. Early proactive management is a must, and dose reduction should be considered as early as grade 1. Patients who develop radiological changes suggestive of non-infectious pneumonitis and those have few or no symptoms may continue everolimus without dose adjustments.

Patients should be advised to report promptly any new or worsening respiratory symptoms, and it is recommended that patients keep a diary to monitor these effects [19]. In addition, patients may consider taking warm baths/showers, or using a vaporiser to help thin out secretions; and avoiding allergens, such as smoke and pollen, if pneumonitis is present [46].

### Infections

While the rates of infections were no higher with everolimus than placebo in the EXIST-1 and -2 studies (around 70% in both groups in both trials) [20, 21], everolimus has immunosuppressive properties and patients may be susceptible to bacterial, fungal, viral or protozoal infections, including opportunistic pathogens and reactivation of previous infections (see Table 1 for a list of described infections). Hence, in normal clinical practice some increase in infection rates may be expected, even in patients not taking other concurrent immunosuppressants. Any pre-existing infections should be treated appropriately and resolved before commencing therapy. Guidelines for hepatitis B screening, prophylaxis and reactivation management have been developed [34].

If a diagnosis of infection is made, appropriate treatment must be given promptly. Everolimus interruption/discontinuation should also be considered in this scenario, particularly when associated with fever. In cases of invasive systemic fungal infection, everolimus should be discontinued and appropriate antifungal therapy initiated [34]. Patients being treated with everolimus should be warned about the risk of infection and educated on the need to seek medical help if they develop suggestive symptoms and if they develop cuts or wounds because these are at an elevated risk of becoming infected. Furthermore, to reduce the risk of infection, patients should wash their hands frequently keep food preparation areas clean, and avoid cleaning up after pets.

### Stomatitis

Stomatitis is an inflammation of the mucous membranes in the oral cavity, inner surface of the lips or the tongue, and is associated with erythema, oedema, a burning sensation and occasionally bleeding. It can impair a patient’s ability to eat, swallow and talk, and can cause significant pain. Stomatitis is one of the most common AEs in patients treated with mTOR inhibitors, although the effect is typically transient. It usually occurs within 1 month of treatment initiation.

The early and active management of stomatitis is essential. When starting everolimus, patients should be issued with suitable oral care products (e.g. sucralfate suspension, bioadherent oral rinse gels, raspberry mucilage). For example, sucralfate suspension can be applied 3–4 times day with a cotton wool bud to the ulcer or patients can rinse and spit. The sucralfate suspension can also be swallowed if the patient has a lesion in the pharynx. The steroid triamcinolone acetonide can be used for ulcers on or inside the lip, or in the front of the mouth [34]. For those who do not respond to these medications, or those who require greater pain control, a topical analgesic (e.g. benzocaine with or without a steroid) may be required. Some clinicians recommend a ‘magic mouthwash’ for which several formulations are available, but which typically contains a topical anaesthetic, a steroid, an antibiotic, an anti-fungal and an antacid – it should however be noted that concomitant anti-fungals are not recommended with everolimus treatment [19]. Direct application of clobetasol, a high potency steroid, has been associated with rapid symptomatic improvement in mTOR-treated patients with aphthous ulceration [47].

All patients with stomatitis should receive recommendations regarding good oral care. This may include consistent regular brushing with a soft toothbrush that is changed on a regular basis, frequent rinsing with bland rinses (e.g. sterile water, saline or sodium bicarbonate), and avoidance of strong-flavoured toothpastes and those containing lauryl sulphate; instead, children’s toothpastes may be favoured. Mouthwashes containing alcohol should be avoided. Patients should also avoid acidic, spicy, hard or crunchy foods, those that are too hot in temperature, and alcohol. Care should be taken when eating and drinking; it may be advisable to eat 5–6 smaller meals per day and drink through a straw. Physicians should also consider vitamin B and zinc supplementation prior to everolimus initiation; while the data supporting the evidence of this in ameliorating mouth ulcers are mixed [48–51], there are likely patients who are both deficient and predisposed to this side effect of mTOR inhibition and who would therefore benefit.

### Rash

Rash in everolimus-treated patients is usually macular or papular, and may also contain pustules and eczematous changes. It is most commonly found on the face, upper trunk and scalp, and may be associated with pruritus. Topical treatments are typically the first choice for managing rash, such as those products containing benzoyl peroxide and an antibiotic, or topical steroids, such as alcometasone or mometasone.

Oral antibiotics (e.g. minocycline or doxycycline) may also be necessary. In patients with pruritus, topical emollients or antihistamines may be advisable. Retinoids should be avoided as they may disrupt skin integrity and potentially increase risk of infection. Physicians should advise patients to consider the following advice: wear loose, comfortable clothing; use mild soaps without perfume and take short, lukewarm showers; when washing and drying, pat the area instead of rubbing with a towel or washcloth; avoid tanning booths; use a moisturiser frequently; and use sunscreen (at least SPF 15) [52].

### Metabolic events

The metabolic events that often occur with everolimus treatment – namely hypercholesterolaemia, hyperglycaemia, hypophosphataemia, hypertriglyceridaemia, elevated lactate dehydrogenase and hyperuricaemia – are shown in Table 2 [19]. Abnormal laboratory values can usually be managed routinely without treatment interruption. Intervention is recommended at grade 3 severity, with the nature of the intervention dependent on the specific metabolic abnormality. It is important to bear in mind that hypercholesterolaemia can increase the risk of cardiovascular events and that hypertriglyceridaemia at levels of ≥1,000 mg/dL can cause life-threatening pancreatitis.

With regard to practical management, fasting blood glucose and lipid profiles should be assessed before starting everolimus and then monitored periodically (e.g. every 6–8 weeks) during treatment. Patients with diabetes require careful monitoring – and potential modification – of their antihyperglycaemic medications. In particular, metformin should be avoided in diabetic patients with renal impairment (creatinine clearance <30 mL/min/1.73 m^2^) and in patients with lactic acidosis [53]. Statins and fibrates are recommended for lowering total cholesterol and triglycerides, respectively, along with lifestyle and nutritional changes. Consideration should be given to the use of lipid regulating drugs since some may not be licensed for use in children.

### Haematological toxicity

Bone marrow suppression is a common toxicity associated with mTOR inhibitors. Grade 1 effects do not require any interruption of treatment. Thrombocytopenia requires intervention at grade 2 and neutropenia at grade 3. Thrombocytopenia and neutropenia are rarely associated with clinically significant bleeding or infection, and hence do not typically necessitate platelet transfusion or growth factor support [54]. Microcytosis and hypochromia have also been reported in patients with TSC treated with mTOR inhibitors; generally these effects are self-limiting [18].

In cases of grade 3 toxicity, interruption of treatment with everolimus is required, with a lowering of the dose upon resumption. Everolimus should be discontinued in any cases of life-threatening toxicity.

### Renal AEs and proteinuria

Renal tubular abnormalities have been observed in patients treated with sirolimus [54], and hence physicians should be vigilant when initiating everolimus, particularly in patients with TSC with significant renal involvement. Renal function should be assessed before starting everolimus treatment and periodically thereafter (Fig. 1), which should include assessment of urinary protein (e.g. urine protein:creatinine ratio). Although AEs such as proteinuria or an increased degree of proteinuria may be common, they are generally intermittent and should not trigger treatment cessation. Angiotensin-converting enzyme (ACE) inhibitors and angiotensin receptor blockers can be used to ameliorate microalbuminuria or proteinuria when necessary. Cessation of everolimus should be considered if there is progressively increasing proteinuria to >1 g/day, especially if >3 g/day or if associated with peripheral oedema. Similarly if GFR progressively declines to <30 mL/min, cessation should be considered, although this may be due to the underlying pathophysiology of TSC rather than everolimus.

### Additional patient considerations

#### Drug interactions

Everolimus is a substrate of cytochrome P450 3A4 (CYP3A4), and of the moderate inhibitor of P-glycoprotein (P-gp). Hence, blood levels of everolimus may be affected by products that inhibit or induce CYP3A4 and/or P-gp. Specifically, CYP3A4 and P-gp inhibitors increase everolimus concentration and inducers decrease everolimus concentration. Many patients with TSC take antiepileptic medications that are CYP3A4 and/or P-gp inducers, which may decrease everolimus concentrations. Indeed, in EXIST-2, mean trough blood concentrations of everolimus were 4–8 ng/mL in patients taking enzyme-inducing drugs (mainly carbamazepine), compared with 8.4–12.3 ng/mL in those who were not [31].

Table 4 provides a list of inhibitors and inducers, and recommendations for co-administration with everolimus [19]. Potent inhibitors of CYP3A4 and/or P-gp are contraindicated with everolimus treatment; moderate inhibitors should be used with caution, when unavoidable. If co-administration of a CYP3A4 inducer (e.g. carbamazepine) is required in patients treated for AML, the everolimus dose may need to be increased up to 20 mg/day. Similarly, patients treated for SEGA may require an increased everolimus dose to achieve the same exposure as patients not taking potent inducers. Dosing should be titrated to attain trough concentrations of 5–15 ng/mL. If concentrations fall below 5 ng/mL, the daily dose may be increased by 2.5 mg every 2 weeks, checking the trough level and assessing tolerability before increasing the dose. Patients should be advised not to use St John’s Wort (a CYP3A4 inducer) or drink grapefruit juice (a moderate CYP3A4/P-gp inhibitor) during treatment with everolimus [19].

**Table 4:** Drug interactions of note with everolimus [19]

Drug type	Recommendation
*Potent CYP3A4 and/or P-gp inhibitors* Ketoconazole, itraconazole, posaconazole, voriconazole telithromycin, clarithromycin, nefazodone, ritonavir, atazanavir, saquinavir, darunavir, indinavir, nelfinavir	Concomitant treatment of everolimus and potent inhibitors is not recommended.
*Moderate CYP3A4 and/or P-gp inhibitors* Erythromycin, imatinib, verapamil, ciclosporin oral, fluconazole diltiazem, dronedarone, amprenavir, fosamprenavir	Use caution when co-administration of moderate CYP3A4 inhibitors or P-gp inhibitors cannot be avoided. *For patients with AML:* If patients require co-administration of a moderate CYP3A4 or P-gp inhibitor, dose reduction to 5 mg or 2.5 mg daily may be considered. However, there are no clinical data to guide this dose adjustment. Due to between-subject variability, the recommended dose adjustments may not be optimal in all individuals; therefore, close monitoring of adverse events is recommended. If the moderate inhibitor is discontinued, consider a washout period of at least 2–3 days before the everolimus dose is returned to the dose used prior to initiation of the co-administration. *For patients with SEGA:* If patients require co-administration of a moderate CYP3A4 or P-pg inhibitor, dose reduction by approximately 50% may be considered. Further dose reduction may be required to manage adverse reactions. Trough concentrations should be assessed approximately 2 weeks after the addition of the CYP3A4/P-gp inhibitor; if this inhibitor is discontinued a 2–3 washout period should be considered before everolimus reinitiation. Everolimus trough concentrations should be assessed approximately 2 weeks after any change in dose.
*Potent and moderate CYP3A4 inducers* Rifampicin, dexamethasone, antiepileptic agents (e.g. carbamazepine, phenobarbital, phenytoin), efavirenz, nevirapine	Avoid the use of concomitant potent CYP3A4 inducers. *For patients with AML:* If patients require co-administration of a potent CYP3A4 inducer, an everolimus dose increase from 10 mg/day up to 20 mg/day should be considered, using 5 mg increments or less applied on Days 4 and 8 following start of the inducer. This dose of everolimus is predicted to adjust the AUC to the range observed without inducers. However, there are no clinical data to support this dose adjustment. If treatment with the inducer is discontinued, consider a washout period of at least 3–5 days (reasonable time for significant enzyme de-induction) before the everolimus dose is returned to the dose used prior to initiation of the co-administration *For patients with SEGA:* Patients receiving concomitant potent CYP3A4 inducers may require an increased everolimus dose to achieve the same exposure as patients not taking inducers. Dosing should be titrated to attain trough concentrations of 5–15 ng/mL; daily dose may be increased by 2.5 every 2 weeks if values are below this, checking the tolerability and trough levels before increasing. If the inhibitor is discontinued a 2–3 washout period should be considered before everolimus reinitiation. Everolimus trough concentrations should be assessed approximately 2 weeks after any change in dose.
*CYP3A4 inducer* St John’s Wort (*Hypericum perforatum*)	Preparations containing St John’s Wort should not be used during treatment with everolimus

*AUC* area under the curve, *CYP3A4* cytochrome P450 3A4, *P-gp* P-glycoprotein

Patients taking concomitant ACE inhibitors may be at increased risk of angioedema, although the evidence is not conclusive, and is seldom seen in practice [19]. Impaired wound healing is a class effect of rapamycin derivatives, including everolimus; caution should therefore be exercised with the use of everolimus in the peri-surgical period [19], and treatment should be stopped 7–14 days prior to major invasive surgery procedures. The treatment may be restarted after the surgical site is completely healed [54].

#### Special populations

No dose adjustment is required in elderly patients (aged ≥65 years) who are otherwise fit, or in patients with renal impairment [19]. In patients with renal AML and hepatic impairment, the dose should be adjusted based on Child–Pugh class [19, 55]. Everolimus dosing should also be adjusted in adult patients with SEGA and hepatic impairment [19, 55]. Everolimus is not recommended for patients <18 years of age with SEGA and hepatic impairment [19]. Calculation of Child-Pugh status and recommended everolimus dosing according to this status is shown in Table 5. Everolimus whole blood trough concentrations should be assessed around 2 weeks after any change in Child–Pugh status [19].

**Table 5:** Calculating Child–Pugh status and everolimus dosage recommendations [19, 55]

Factor	1 point	2 points	3 points
Total bilirubin (μmol/L)	<34	34–50	>50
Serum albumin (g/L)	>35	28–35	<28
PT INR	<1.7	1.71–2.30	>2.30
Ascites	None	Mild to moderate	Severe/refractory
Hepatic encephalopathy	None	Grade I–II (or suppressed with medication)	Grade III–IV (or refractory)
Child–Pugh class	Class A	Class B	Class C
Total points	5–6	7–9	10–15
Recommendation in patients with AML	The recommended dose is 7.5 mg daily	The recommended dose is 5 mg daily	Everolimus is only recommended if the desired benefit outweighs the risk; a dose of 2.5 mg daily must not be exceeded
Recommendation in patients with SEGA	75% of the recommended starting dose, calculation based on BSA (rounded to the nearest strength)	25% of the recommended starting dose, calculation based on BSA (rounded to the nearest strength)	Everolimus is not recommended

*PT INR* prothrombin time and international normalized ratio

#### Monitoring blood levels of everolimus

In patients treated for SEGA, therapeutic drug monitoring of everolimus blood concentrations is a requirement, using a validated assay [19]. In patients aged <3 years of age, trough blood concentrations should be assessed ≥1 week after commencing treatment; for patients aged ≥3 years this should be done at around 2 weeks after commencing treatment. Once a stable dose is attained, trough concentrations should be monitored every 3–6 months in patients with changing BSA or every 6–12 months in patients with stable BSA, for the duration of treatment.

In patients treated for renal AML associated with TSC, therapeutic drug-monitoring of everolimus blood concentrations is an option that should be considered, using a validated assay [19]. Although the therapeutic range of everolimus has not yet been defined, the blood levels achieved with any particular dose varies between individuals; trough concentrations in EXIST-2 ranged from 7.63 to 9.37 ng/mL with large inter-individual variability [20]. A subsequent pharmacokinetic/pharmacodynamic analysis found that trough levels ranged from 4.57 to 13.10 ng/mL for patients receiving either 5 mg or 10 mg everolimus [31]. In an open-label extension of EXIST-1, the median dose intensity per day was 5.9 mg/m^2^ with a range of 1.0–13.7 [23]. Therefore, monitoring may be useful after initiation or a change of everolimus dose/pharmaceutical form. Monitoring should also be considered when CYP3A4 inducers or inhibitors are prescribed, or in cases in which there is a change in hepatic status (e.g. Child–Pugh status; Table 5) [19]. When performing blood tests, consider using EMLA cream, a local anaesthetic cream, or a freeze spray to minimise discomfort at the site of injection.

#### Fertility

An important consideration of treatment with everolimus is fertility and how treatment may affect the ability of the patients to conceive. According to the everolimus Summary of Product Characteristics, women of childbearing potential are advised to use a highly effective method of contraception for the duration of treatment with everolimus and for up to 8 weeks after the cessation of treatment [19]. It is the opinion of the authors that women should cease everolimus ≥2 weeks before stopping contraception — men may be allowed to continue everolimus while attempting to father children; however, if unsuccessful a discussion may be needed about treatment cessation. Additionally, it may be prudent for patients to have eggs or sperm stored if they wish to have children after treatment has finished.

## Conclusions

Although everolimus-related AEs are fairly common in patients with TSC they are often minor and usually transient. More serious or recurrent AEs can often be managed through dose adjustment or temporary cessation of therapy. It is important to recognise, however, that the careful management of AEs means that patients can continue on therapy for longer in order to maximise benefit. Indeed, randomised studies of everolimus in patients with TSC have shown that despite the AEs that can occur, overall the benefits outweigh the risks. In the EXIST-2 trial of TSC patients with AML, 5% (3 of 79) of everolimus-treated patients discontinued due to AEs, whereas 42% (33 of 79) responded [20]. In the EXIST-1 trial of TSC patients with SEGA, no everolimus-treated patients discontinued due to AEs, whereas 35% (27 of 78) responded to treatment [21]. Additionally, expanded-access trials in real-world patients with AML or SEGA support the conclusion that everolimus has a manageable safety profile [56, 57].

The active management of TSC is important when prescribing everolimus. Physicians should anticipate AEs such as rash and stomatitis, and take appropriate measures to prevent them worsening for those affected. Figure 1 shows a protocol that may prove useful to physicians throughout the management of patients with TSC.

Physicians must be prepared to provide early education to the patient (or carers/parents in the case of paediatric patients) to aid this management, and in the case of patients with learning difficulties must consider additional strategies to deal with AEs (Table 6). In addition to a list of items to consider prior to the initiation of treatment with everolimus dosing as well as monitoring requirements are also important. As TSC is treated by a number of specialists such as nephrologists, neurologists, paediatricians and geneticists, it is important that the multi-organ effects of the disease and AEs are considered in any management programme.

**Table 6:** Information for patients

*Infection* • Patients should be advised that they may be more susceptible to infections during treatment with everolimus, and to be aware of and to promptly report to a healthcare professional any signs and symptoms of infection, including a raised temperature *Stomatitis* • Brush teeth regularly and gently with a soft toothbrush • Use a mild toothpaste (e.g. children’s toothpaste) • Rise frequently with a bland mouthwash, such as water or a salt mouthwash (e.g. half a teaspoon of salt in a cup of warm water) • Avoid mouthwashes containing alcohol • Avoid hot food (in temperature and/spiciness) or crunchy food *Non-infectious pneumonitis* • Patients should be advised to promptly report any new or worsening respiratory symptoms *Rash* • Use a mild, unperfumed soap • Wear loose convertible clothes • When washing and drying the body, pat dry instead of rubbing with a towel • Moisturise frequently • Take short, lukewarm showers • Use sunscreen (at least SPF 15)	

Continued surveillance is also required for possible longer-term concerns, such as suppression of fertility, proteinuria and hypophosphataemia, because patients with TSC may need indefinite mTOR inhibition due to the fact that tumours tend to regrow upon discontinuation [1]. As with other AEs, these longer-term concerns require active monitoring and management to ensure that patients can continue to receive the benefit of everolimus therapy. Initiatives such as TOSCA (TuberOus SClerosis registry to increase disease Awareness) are underway that aim to provide long-term data on the natural history and treatment of TSC [58], which will no doubt prove useful for the development of future strategies for AE management.

## Abbreviations

AE: Adverse events
AML: Angiomyolipomas
BSA: Body surface area
CYP3A4: Cytochrome P450 3A4
GFR: Glomerular filtration rate
LAM: Lymphangioleiomyomatosis
P-gp: P-glycoprotein
SEGA: Subependymal giant astrocytoma
TSC: Tuberous sclerosis complex
